# Factors influencing integration of TB services in general hospitals in two regions of China: a qualitative study

**DOI:** 10.1186/1472-6963-12-21

**Published:** 2012-01-25

**Authors:** Guanyang Zou, Xiaolin Wei, John D Walley, Jia Yin, Qiang Sun

**Affiliations:** 1 Nuffield Centre for International Health and Development, University of Leeds (based in China), Room 1220, No. 1032 Dongmen North Rd, Luohu District, Shenzhen, 518003, China; 2 The Chinese University of Hong Kong, 2/F, School of Public Health and Primary Care, Prince of Wales Hospital, Shatin, N.T., Hong Kong, China; 3 Nuffield Centre for International Health and Development, University of Leeds, 101 Clarendon Rd, Leeds, LS2 9LJ, UK; 4 Center for Health Management and Policy, Shandong University, China, Mailbox 128, No 44 Wenhua Rd, Jinan, Shandong, 250012, China

**Keywords:** TB control, service delivery, integration, hospitals, China

## Abstract

**Background:**

In the majority of China, the Centre for Disease Control (CDC) at the county level provides both clinical and public health care for TB cases, with hospitals and other health facilities referring suspected TB cases to the CDC. In recent years, an integrated model has emerged, where the CDC remains the basic management unit for TB control, while a general hospital is designated to provide clinical care for TB patients. This study aims to explore the factors that influence the integration of TB services in general hospitals and generate knowledge to aid the scale-up of integration of TB services in China.

**Methods:**

This study adopted a qualitative approach using interviews from sites in East and West China. Analysis was conducted using a thematic framework approach.

**Results:**

The more prosperous site in East China was more coordinated and thus had a better method of resource allocation and more patient-orientated service, compared with the poorer site in the West. The development of public health organizations appeared to influence how effectively integration occurred. An understanding from staff that hospitals had better capacity to treat TB patients than CDCs was a strong rationale for integration. However, the economic and political interests might act as a barrier to effective integration. Both sites shared the same challenges of attracting and retaining a skilled workforce for the TB services. The role of the health bureau was more directive in the Western site, while a more participatory and collaborative approach was adopted in the Eastern site.

**Conclusion:**

The process of integration identifies similarities and differences between sites in more affluent East China and poorer West China. Integration of TB services in the hospitals needs to address the challenges of stakeholder motivations and resource allocation. Effective inter-organizational collaboration could help to improve the efficiency and quality of TB service. Key words: TB control, service delivery, integration, hospitals, China.

## Background

### Integration of TB services into general health services

Tuberculosis (TB) control is of great public health importance, particularly in many low-and middle-income countries. The World Health Organization (WHO) TB strategy, brand named "DOTS", is based on five essential elements: political commitment; case detection using sputum microscopy; standardized short course chemotherapy; regular drug supply; and monitoring and evaluation [1]. WHO and International Union against TB and Lung Disease (IUATLD) have strongly recommended that TB and other disease control programmes should be closely integrated within wider health systems. However, in many countries national TB programmes function vertically as part of the communicable disease control system, operating outside the existing health care structures [2]. Raviglione and Pio suggest that this integration should be through "a pragmatic approach combining a specialized, well defined management system with a fully integrated service delivery" [3]. Unger et al defines integration as "a process where disease control activities are functionally merged or tightly coordinated with multifunctional health care delivery" [4]. In particular within this paper, integration is used to describe the inclusion of TB control activities, especially clinical services, into general health services.

Integration of TB services into primary health care has been common since the 1970s [5], however this has been less successful within secondary health care. Involving public and private hospitals for effective TB programme delivery is part of WHO's Stop TB strategy called Public-Private Mix (PPM)-DOTS [6]. Although integration of TB services within public and private hospitals in many African countries has been achieved, this has been less successful in parts of Asia where hospitals were not included in initial plans for rapid TB service expansion [7]. Indonesia is one example of an Asian country where DOTS is increasingly included within public and private hospitals [8-11]. Despite the successful scale-up, hospitals have reported lower cure and success rates, compared to chest clinics and health centres [8], with a large proportion of patients not treated with standardized diagnosis and treatment [9]. In order to strengthen integration of DOTS within hospitals, a combination of strong individual commitment of health professionals, organizational supports, partnership governance, and relevant policy is required [9].

### TB control in China

China has one of the highest TB burdens in the world, with more than 1.3 million new cases each year [12]. The Centre for Disease Control and Prevention (CDC) at various levels was established during the national health sector reform since early 2000, by incorporating previous anti-epidemic stations and related public health programmes [12]. Accordingly, many TB dispensaries were merged into the CDC as an affiliated department or a department combined with other public health programmes. The national CDC, under the leadership of the Ministry of Health, provides technical support and routine management of disease control programmes, including the National TB Programme (NTP). This structure is replicated at provincial, prefecture and county levels [13].

Before 2003, TB control performance was poor, with detection of smear-positive TB at around 30%[12]. The inadequate control of TB was related to a malfunctioning health system, which resulted from the transition to a market economy and financial decentralization in the mid-1980s [14]. Since then, health funding and management responsibilities were decentralized from central and provincial governments, to county and township governments [15]. Due to diminished government funding, both the hospital sector (largely public) and public health organizations had to rely on providing additional services and selling drugs to subsidize their total income [14]. During this time, TB control, as with many other public health activities, was neglected as it was labor intensive and created little income [12]. Where TB diagnosis and treatment was provided for free, many CDCs charged patients for additional tests and drugs, often with uncertain benefit [12,16].

China's TB control has benefited from a series of measures to strengthen the public-health system following the outbreak of severe acute respiratory syndrome (SARS) in 2003 [12]. Since 2003, there has been significantly increased funding for public health, especially for TB. In 2004 the law on the control of infectious diseases was revised, and the national internet-based communicable disease reporting system was implemented to ensure cases of TB are reported. By 2005, China achieved the global targets for TB control, with complete DOTS coverage, 80% of new smear-positive TB case detection, and over 90% treatment success rate [12].

### Integration of TB services: from the CDC model to the integrated model

In the majority of China, the county CDC provides both clinical (diagnosis, treatment and clinical management) and public health functions (education, supervision and reporting) for TB cases. General hospitals and other health facilities such as community-based health services are required to refer suspected TB cases with cough of two weeks or more or haemoptysis to the CDC. Only complicated and severe TB cases are treated in the general hospitals. The CDC traces all referred cases not visiting the CDC within three days. Only TB patients registered and treated in the CDC are entitled to free treatment under the government's TB programme. This model is known as "CDC model".

This model to TB services has come across a number of challenges. Thirty-four percent of patients with a TB diagnosis first presented at the general hospital system after the onset of their symptoms. However, only 25% of these cases were reported and 13% referred to the CDC [17]. As a result, a large proportion of patients have not been seen within CDC DOTS services. Another concern is the low adherence to TB diagnosis and treatment guidelines in hospitals, with treatment often including unnecessary prescriptions [18]. In addition, the CDC has limited staff and clinical capacity [19], particularly after the enactment of the Law for Licensing Medical Practitioners which states that public health doctors are not eligible to provide clinical care.

Since early 2000s an integrated model of TB service delivery was implemented in some eastern provinces such as Shanghai, Zhejiang and Jiangsu, on their own initiatives and some of the western provinces with support from the Global Fund to Fight AIDS, Tuberculosis and Malaria (GFATM). In this model, the CDC continues to provide public health functions such as referral management, reporting, staff training and case tracing. A general hospital was "designated" to undertake diagnosis, prescription and follow-up management, with other hospitals and health facilities referring those suspected of having TB to the designated hospital. A comparison of the TB functions between the two models is summarized in Table 1.

**Table 1:** Comparison of TB functions between the CDC and integrated model

Functions	The CDC model	The integrated model
Identify suspected cases of TB	All health facilities	All health facilities

Diagnose and confirm TB	CDC	Designated hospital

Prescribe treatment for TB	CDC	Designated hospital

Perform clinical follow-up	CDC	Designated hospital

Record and report cases	CDC	Designated hospital

Supervise treatment or assign treatment observer	Community-based health facilities	Community-based health facilities

Follow up on defaulters, train hospital staff, supervise networks, laboratory EQA, monitor and evaluate, provide supplies and medicine	CDC	CDC

NB: In the both models, community-based health services, ie, township hospitals and village clinics play an important role in TB control. However, their roles are only limited on referring the TB cases or suspects to the CDC or the designated hospital, supporting the CDC to conduct the defaulter tracing, and providing treatment supervisions for the TB patients.

To date, a few studies in China have reported the experience of integrating TB services in the township hospitals (functioning as health centers) of Guangxi [20,21], Shandong [22,23] and Gansu provinces [24]. However, there is a lack of published evidence on the integration of TB care in the general hospitals. This study aims to explore the factors that influence the integration process and generate knowledge for the scale-up of the integrated model in China. It is one of the first few studies which research the integrated model in China. This study will not only provide evidence for the implementation and scale-up of the new policy in China, but also adds to the currently limited research on integration of TB service in the larger public or private hospitals in similar settings.

## Methods

This study adopted a qualitative approach, particularly valuable when seeking to explore processes of implementation [25].

### Research sites

In order to better understand the drivers and barriers of policy implementation, we chose sites from different economic development regions for this study, i.e. from the East and West provinces. Although GFATM supported districts might exist, we purposefully selected districts which initiated the integrated model on their own initiative, as a bottom-up approach was thought to be more replicable and sustainable. At the time of this study, very few districts in China had implemented the integrated model. Thus research sites were pragmatically selected, considering the availability of the sites and their willingness to participate. Finally, two districts were selected from the database of the China National TB Programme. One was Changning, a district of Shanghai (SC), and another was Nanning, the capital city of Guangxi(GN). Geographically, they are located in eastern and south western China respectively. SC had much higher GDP per capita and TB expenditure per person than GN. Both sites had similar cure rates of new smear positive TB cases (Table 2). A prefectural general hospital in GN provided TB services since 1996 but it was not until 2005 that TB services were fully integrated in this hospital. In SC, TB services were integrated in a district general hospital in 2007. Under the leadership of health bureaus, the designated hospitals in both sites work with the CDCs at the same levels, and other general health facilities to deliver TB service within their catchments areas.

**Table 2:** General social economic situations and health financing for TB in Shanghai Changning and Guangxi Nanning (2007)

Indicators	Shanghai Changning	Guangxi Nanning
Per capita GDP (RMB)	88,785	15,774
TB expenditure per person (RMB)	0.99	0.42
Notification rate of TB patients (per million)	322	999
Cure rate of new smear positive TB cases (%)	88.5	89.8
Number of general hospitals in the district	10	46

### Data collection

Purposive sampling techniques were employed to select participants based on their disciplines and roles. These individuals represented a wide spectrum of health professionals working within TB control. 21 individual interviews were conducted, 11 at SC and 10 at GN, in the autumn of 2008 (Table 3). The interviewees included: directors of the CDC, the designated hospital, and another general hospital; CDC staff responsible for the service delivery; and practitioners from departments of the laboratory, outpatient, and patient wards of the designated hospital and another general hospital. A semi-structured topic guide was developed and used for the interviews, covering topics including background and history of integration, and process of integration, including health financing, leadership, service delivery, task mix and communication.

**Table 3:** Sampling for the in-depth interviews in Shanghai Changning and Guangxi Nanning

Organisation	Positions	Shanghai Changning	Guangxi Nanning
CDC	Director	1	1
	TB section chief	1	1

	Director	1	1
	Outpatient doctor	2	1
Designated hospital	Inpatient doctor	1	1
	Laboratory staff	1	1
	Radiology staff	1	1
	Public health staff	1	1

Other general hospital	Directors	1	1
	Outpatient staff	1	1

Total		11	10

The interviews were conducted by a core research team, mainly consisting of TB researchers from the University of Leeds based in China (XW, GZ and JY) and Shandong University (QS). One member was responsible for ensuring consistency throughout the data collection process. Interviewees were invited for interview through the CDC and appointments made via telephone or Email, outlining the purpose of the research and questions to be asked. Informed consent was obtained prior to the interviews. Each interview lasted approximately 40-50 minutes, and was audio recorded with permission of the interviewees. The recording was transcribed within 48 hours by trained postgraduate students from Shandong University and checked by at least one core research team member. This ensured that researchers could return the unclear points of the transcripts to the interviewees for clarification in a timely manner. Ethical approval was granted by the Ethical Committee of School of Public Health of Shandong University.

### Data analysis

A thematic approach was used, which allows both application of an existing framework and inclusion of further emerging themes from the data [25]. All the data sources were imported into the Weft QDA, a computer-assisted qualitative analysis tool. The topic guides and transcripts were scrutinized to identify emerging and recurrent themes and a framework table was progressively established and structured. These themes and sub-themes were input into the tree-structure of Weft QDA. Transcripts of each interview were read through and coded into the related themes and sub-themes. Coding was an iterative process which permitted to revise the existing themes and include emerging themes from the data. Table 4 illustrates examples of the coding process. The analysis was carried out by two different persons within the research team (one conducted the initial analysis, while another checked the appropriateness of the coding and categorizing). A structured, focused approach was used which helped to identify similarities and differences across the two sites [25].

**Table 4:** Examples of the coding process (translated)

Transcripts	Condensed meaning	Sub-themes	Themes
"Our doctors normally would not admit the general TB patients and referral of severe TB patients is their responsibility. If we admit the general TB patients, other patients will complain. Especially the migrant patients, [who] do not have medical insurance so they can't be reimbursed for the inpatient cost". (SC, hospital staff)	"Doctors normally not admit the general TB patients, referral of severe TB patients, other patients will complain admitting the general TB patients, migrant patients did not have medical insurance."	impact of resource allocation on hospitalization (patient-centredness, public health approach)	resource allocation

"The health bureau organizes 4-5 special meetings for the CDC and designated hospital annually. The coordination from health bureau is effective. For example, the hospital tried to minimize the distribution of the case management allowance (to community doctors) and it was the health bureau that solved the problem. Our coordination with hospital is generally getting better and better." (GN, CDC staff)	"Health bureau organizes 4-5 special meetings annually, coordination from health bureau effective, hospital minimized case management allowance, health bureau solved problem, coordination better and better."	role of health bureau relationship between CDC and hospital	management coordination

### Validity and reliability of the study

In qualitative research, participant and researcher bias may affect the validity and reliability of findings. Efforts were made to overcome the sources of biases which could derive from questions design, sampling, data collection and analysis [27]. Working in a team for the data analysis provided a form of triangulation [27-29]. Another two members who were not involved with data analysis revisited the emerging themes and refined their relationships. Another member of research staff, not involved with this study, was invited to check for the consistency of the original themes and the themes arising from his understanding. Any discrepancies arising within the team and between the team and the external reviewer were addressed through the consensus. This process in return improved our insight of the original context and understanding of the transcripts.

## Results

Seven categories of factors which influenced TB service integration were: historical context, clinical capacity, motivation for integration, resource allocation, incentive and staffing, management coordination, and technical exchange.

### 1. Historical context

In both sites, the integration process was found to be contingent on the restructuring process of the public health organizations (Table 5). This restructuring process reflected the continuous health sector reform under the context of financial decentralization in China, including the establishment of the CDCs in early 2000. This whole process had witnessed the weakening of the clinical functions of the CDCs and redeployment of the TB control staff to the designated hospitals. Since then, TB services were integrated into the designated hospital. This process coincided with the enactment of the Law for Licensing Medical Practitioners in the late 1990s, which made public health doctors become ineligible to provide clinical care.

**Table 5:** Organizational transition in Shanghai Changning and Guangxi Nanning

Shanghai Changning	Guangxi Nanning
In SC, there was an independent TB dispensary directly affiliated to the health bureau before 1995. However, the development of TB dispensary was challenged by the diminished government funding. In 1995 a chronic Disease Control Institute was established and TB dispensary was merged into this institute. In 2000, following the national health sector reform the CDC was set up and the chronic disease control station was merged into the CDC. In 2007, the CDC was moved into a new public health building, where conducting TB clinical activities seemed no longer appropriate. By that time, most of the other districts in Shanghai had adopted the integrated model. At the request of the health bureau, a district general hospital was designated to provide TB service.	In GN, early in 1996, TB clinical service was co-provided by an independent TB dispensary, a prefectural general hospital, a medical university affiliated hospital, a provincial hospital. However, it was recognized that the complexity of TB service delivery structure had resulted in the poor coordination and management of TB care. In 2002, the prefecture CDC was established following the national reform, incorporating the anti-epidemic station, health education institute and the TB dispensary. In 2005, the prefectural general hospital was designated to fully provide the TB service, and all the TB cases/suspects were required to be referred to this hospital for standardized treatment and management.

### 2. Clinical capacity

The capacity of CDC staff to treat TB patients remained challenging, reflecting the practical need for such integration. For example, the doctors in GN's designated hospital criticized the CDC doctors' lower clinical capacity, and poorer knowledge on complications such as liver damage and respiratory failure. The CDC staff seemed to agree with such criticism and suggested that integration could make the best of the public health strength of the CDC and the clinical strength of the hospital.

"It was difficult for us to treat TB patients, with limited technology, equipment and staff. We are especially not confident in treating the complications. After the clinical function was transferred to the hospital, we could focus more on management... We are public health doctors so we should do more on epidemiology and health education." (GN, CDC staff)

### 3. Motivation for integration

The initial integration process faced some barriers. It was apparent that there was a lack of motivation of staff to move towards the integrated model within the CDCs. Interviews suggested that political will and economic interests would be potential barriers to integration. One member of CDC staff in SC expressed they favored the CDC model, which allowed them to better control the affiliated TB clinic. The CDC staff from GN reported that they did not want integration initially, complaining this would mean "sacrificing" their service.

"We did not agree to take this approach initially. Although treatment of the smear-positive patients was free, we could still charge from the smear-negative patients. We (CDC) were just set up at that time, with very constrained funding. We could have supplemented the funding through TB treatment. We made great sacrifice through the integration initiative. At that time, we were located in the same yard with the hospital. Both hospital staff and we (CDC staff) waited outside the doors to 'compete' for TB patients (laugh...) But we gradually accept this reality with the increasing funding from the government." (GN, CDC staff)

Motivation for integration appeared to be mixed within the two hospitals. GN's hospital began to treat TB in 1996. Some recalled they did not want to take over the full TB service as the funding was rather limited to support their activities. On the other hand, some seemed to be happy with full integration as they thought treatment of TB could improve their reputation and increase the hospital revenues. In SC, a smooth transition process was observed in the hospital. By 2000, other districts of Shanghai had set up the TB clinics in the general hospital which may have motivated the local hospital to take over the TB service. In addition, as a designated hospital for other infectious diseases such as hepatitis, they had already established a good relationship with the CDC. Lastly, the health bureau agreed to allocate the related TB control budget, previously allocated to the TB clinics of the CDC, to the TB clinics within the designated hospital, with staff costs met by the district government. These factors are likely to have contributed to the smooth transition in SC.

### 4. Resource allocation

In GN, the hospital received limited funding from the health bureau from 1997 to 2003. Since 2004, it received project funding from the Ministry of Health (MoH), matched by local government. However, some felt that this funding was too limited to cover costs of service operation and were concerned about the approach to budget allocation which was often reallocated from the CDC.

"We have to pay extra money to buy a mini-car to conduct patient supervision activities and pay the associated costs from our general budget. We also need to subsidize the associated staff and electricity costs to conduct the X-ray service for TB diagnosis. The more we do, the more we have to pay from our own pocket... I believe there will be further support from the government when the Project comes to a close but the key issue is how to allocate the TB control budget to the hospital in the most effective way." (GN, hospital staff)

This difficulty with funding may have contributed to the hospital attempting to make additional money from treating TB patients, such as prescription of unnecessary drugs and hospitalization. Although staff suggested that only serious TB cases were admitted, the estimated annual hospitalization rate of the TB patients was up to 20%. CDC staff were concerned that this would increase the financial burden of TB patients and may lead to multi-drug resistant TB due to the improper treatment. However, CDC staff were just sympathetic to the hospital's financial dilemma:

"Hospitals are not fully funded by the government; it is understandable to have a higher hospitalization rate but they just admit too many. But what can we do? We could only open an eye and shut an eye as long as the patients are willing to afford. See, hospital directors always mention money." (GN, CDC staff)

In SC, service operation has benefited from a more sustainable resource allocation system. The health bureau directly allocated the operational budget to the designated hospital and funded the TB clinical staff. The hospital mandated that severe TB patients should be referred to the specialist lung hospital or other infectious disease hospitals for treatment. Since funding was not a major concern, there was little sign of unnecessary hospitalization of TB patients. Rather, interviews revealed a more friendly and patient-orientated service provision in SC:

*"Our doctors normally would not admit the general TB patients and referral of severe TB patients is their responsibility. If we admit the general TB patients, other patients will complain. Especially the migrant patients, [who] do not have medical insurance so they can't be reimbursed for the inpatient cost"*.

### 5. Incentives and staffing

Interviews in both sites identified a difficulty in attracting and maintaining skilled TB staff. In both sites, both the doctors and nurses rotated from the infectious disease or respiratory departments on a regular basis. There was serious concern over staffing turn-around and its effect on quality of service:

"We are anxious about the quality of service since no doctors are willing to work in the TB clinics for a longer term. The performance is satisfactory now, but there is crisis in the long run." (SC, CDC staff)

The perceived reasons for an unstable TB team in the hospital included a concern about payment and the potential transmission of TB. For example, TB doctors in both sites thought they did not receive fair pay from the hospital regardless of their qualifications and experiences and the potential transmission risks from TB. They attributed the unfair pay to the fact that the performance-based payment system was not allowed in the TB clinics:

"I feel unfair as a senior physician since there was no major difference for the payment between staff working in the TB department." (GN, hospital staff)

"TB clinical staff received a 'bonus' from the respiratory department. The amount was much less than that for staff working in the wards, albeit they had the same qualifications and experience." (SC CDC staff)

Other reported reasons for an unstable TB team included poor infrastructure, poor job prospects and lack of "preferential" policy (i.e., special incentives) for the staff working in the communicable disease control of the hospital.

"It is not possible to talk about the preferential policy. See, our clinical building was the shabbiest in the hospital, without a waiting room. Most of the patients wait under the tree; other departments at least have waiting rooms." (GN, hospital staff)

"The infectious disease allowance was too humble to attract medical staff to work for the TB clinic. It was the shrinking job market that brought young graduates to work here, neither the payment nor promising future of the TB work." (GN, hospital staff)

### 6. Management coordination

In both sites, an integration hospital leadership group was established including leaders and experts from the health bureau, CDC and hospitals. This group met regularly to discusses the strategic issues, service coordination and emerging problems of TB control.

In SC, some CDC staff thought such official meetings could not address problems effectively. They seemed to less rely on formal meetings and the health bureau to solve problems, but more on personal relationships:

"Only for the bigger issues, we would invite the health bureau to coordinate. Once we ruin the personal relationship with the hospital, nothing can be done."(SC, CDC staff)

In GN, staff also agreed personal relationships were important, but they emphasized a lot on the official coordination from the health bureau which was deemed effective to solve problems. As reported earlier, there was a lasting competitive relationship between the hospital and the CDC due the economic reasons. The health bureau might have thus worked as an effective mediator between the two parties.

"The health bureau organizes 4-5 special meetings for the CDC and designated hospital annually. The coordination from health bureau is effective. For example, the hospital tried to minimize the distribution of the case management allowance (to community doctors) and it was the health bureau that solved the problem. Our coordination with hospital is generally getting better and better." (GN, CDC staff)

### 7. Technical exchange

In GN, TB staff in the hospital attended the general TB training sessions organized by the CDC. The hospital also organized five to six training workshops for the TB-related staff per year. In SC, however, more collaborative and participatory approaches were observed. At the district level, the CDC organized a multi-disciplinary group of TB-related health professionals to discuss TB diagnosis and the quality of x-ray examinations on a weekly basis, including doctors from the respiratory and radiological departments of the designated hospital, community health centers and other general hospitals. This approach was likely to have filtered down from higher level CDC working culture, where the CDC at the municipal level organized multi-disciplinary consultation meetings on the monthly basis. These meetings allowed TB doctors from all the designated hospitals to exchange their experiences of TB diagnoses and treatment. The hospital staff in SC also felt that they received sufficient technical support from the district and municipal CDC during the initial period of integration:

"The public health doctor of the district CDC came to guide our sputum test once per month in the first six months. Experts of the municipal CDC also came to check the quality of smears and sputum culture (three times)." (SC, hospital staff)

The similarities and differences of the two sites in the process of integration are summarized in Table 6.

**Table 6:** Factors influencing the integration of TB services in Shanghai Changning and Guangxi Nanning

Themes	Shanghai Changning	Guangxi Nanning
Historical context	Integration associated with the restructuring process of the public health organizations, especially the establishment of CDC Pressure from enactment of the Law for Licensing Medical Practitioners.	Integration associated with the restructuring process of the public health organizations, especially the establishment of CDC Pressure from enactment of the Law for Licensing Medical Practitioners.

Clinical capacity	Limited capacity of the CDC staff to treat TB cases: rationale for integration	Limited capacity of the CDC staff to treat TB cases: rationale for integration

Motivation for integration	*CDC:* Worried about losing "control" of services: a potential barrier	*CDC:* Worried about losing "profits" of services: a potential barrier
	*Hospital:* Strong budget commitment from health bureau may motivate providers Historical influence: already established good relationship with the CDC before integration Environmental trigger: most of the other districts in Shanghai had adopted the integrated model	*Hospital:* Little budget commitment from health bureau may demotivate providers, but integration perceived to improve reputation and income Historical influence: involving TB service provision since 1996

Resource allocation	More sustainable resource allocation system: health bureau directly allocated operational budget to designated hospital and funded TB clinical staff More patient-centered and public health orientated care: may improve the quality of integration	Funding for the designated hospital rather limited, resources reallocated from CDC to designated hospital; Tendency to hospitalize TB cases, more profit and clinical-orientated care: may affect the quality of integration

Staffing and incentives	Attracting and maintaining skilled TB staff a challenge	Attracting and maintaining skilled TB staff a challenge

Management coordination	Leadership mechanisms in place Personal relationships count	Leadership mechanisms in place Official coordination from the health bureau counts

Technical exchange	Participatory and collaborative approach: may help to improve the quality of integration	Traditional training approach: may limit the quality of integration

## Discussion

### Summary of findings

This study aimed to investigate the factors that influenced the process of integration of TB service from health care professionals' perspectives. The development of public health organizations and the enactment of the Law for Licensing Medical Practitioners appeared to influence how effectively integration occurred. In addition, an understanding by staff that hospitals had better capacity to treat TB patients than CDCs was a strong rationale for integration. However, the economic and political interests might act as a barrier to effective integration of TB control. The more prosperous site (SC) in Eastern China was more coordinated and thus had a better method of resource allocation and more patient-orientated service, compared with the poorer site (GN) in the West. Both sites shared the same challenges of attracting and retaining a skilled workforce for the TB services. The role of the health bureau was more directive in the poorer West, while a more participatory and collaborative approach was adopted in richer East China.

### Policy implications

Our results adds to existing research on factors that influence integration of TB into hospitals [8-11]. These factors reflected the importance of motivation, resources and collaboration in the process of integration, although historical context may play an important role in shaping change [26]. These findings may have important policy implications for the implementation and scale-up of the integrated model in China or other similar health care settings.

The TB programme has long been working in parallel with the general health services. Integration not only involves the shift from a specialized organization to a horizontal one, but also a real transfer of responsibilities, rights and duties [30]. In order to achieve this, challenges which affect the motivation of both the CDC and hospital staff for integration should be addressed. Within the CDC, it is important to address the concern arising from the "loss" of the TB service. The CDC staff in SC worried about "losing control and power" over the vertical service, as operating the service directly was thought to be easier than collaborating with the horizontal health service [30]. In GN, the "loss" of the TB service meant losing profit for CDC. This suggests that under-resourcing of the CDC may have been a barrier to supporting integration, particularly true in West China where costs were not fully met by the local government [15,16,18,22]. Within the hospital, historical involvement in TB services, good relationships with the CDC and an encouraging integration environment prior to integration may help to improve the process of integration. Integration itself can sometimes provide positive incentives since provision of TB service may improve reputation and income for hospitals [9]. However, experiences from the two sites suggest a strong political commitment from health bureau is the key to motivate and engage the hospitals.

Resources are vital to ensure successful service integration [11,31]. The hospital in SC appeared to have more patient-centered care, while the hospital from GN appeared to be more profit-oriented. It is suggested that these differences in mentality were due to differences in resource allocation systems, and security of funding. Following the financial decentralization, performance-based payment systems have been prevalent in Chinese public hospitals, which may have encouraged gaming within the health system to raise additional funds by charging from patients [32]. This approach is not encouraged in the TB clinics, however it is entrenched within practice as operational costs are not fully met by the government. Loss of income due to free TB treatment policy may be a universal concern [11]. Hospital providers may thus comprise the standardised TB treatment through unnecessary prescription of drugs, tests and admissions for general TB patients. These distorted incentives constrain the patient-orientated care provision and can negatively impact on the care of patients. While government funding has largely improved for public health facilities such as CDC, there is an urgent need to increase funding to subsidize the newly integrated public-health services in the hospital. The resource allocation mechanisms in TB control seemed to favor the CDC, and so hospitals may suffer from a "reallocation" of the TB control budget from the CDC. Integration requires transfer of funds for care from the CDC to the hospital. An appropriate budgeting mechanism needs to be explored to ensure equitable allocation of resources and successful integration as in the case of SC.

Different practices of hospitalizing TB patients in SC and GN could also reflect the conflicts between the clinical and public health perspectives of TB care delivery. Clinicians in the hospital should be educated to improve the public health awareness, and specifically implement an outpatient-based systematic public health TB approach. Both sites presented the challenges of retaining qualified TB personnel. To ensure that TB services are sustainable and maintain a high quality of care, fair pay should be provided to attract and stabilize a motivated and skilled workforce.

Successful integration relies on effective inter-organisational collaboration, which could contribute to the efficiency and the quality of TB services by combining resources and expertise from different organizations [33]. Recent work in Indonesia suggests that strengthening the collaboration between the TB programme and hospitals by involving hospitals in DOTS implementation has potential to improve TB control [8-11]. Following the integration, the hospital has changed its role from "reporting and referral" to clinical service provision, while they are well placed to do so [13]. Integration means making the best of the public health strength of the CDC and clinical strength of the hospital, which requires a higher degree of inter-organizational collaboration to ensure successful integration. A more collaborative and participatory inter-organizational approach was observed in the more affluent Eastern China site. This was probably due to a participatory learning culture and the historically better relationship between the designated hospital and CDC. In the poorer Western China site, however, the CDC and hospital had long-lasting competitive relationship often due to challenges in resource allocation and poor communication. Such competitive relationship may fuel a degree of mistrust, thus damaging the collaboration [11]. Experiences from both sites highlight the importance of dialogue and flexibility [34,35], informal communication [11,36], and good governance [11,34] to sustaining such collaboration. A powerful intermediary actor, such as health bureau can play a positive role in mediating such collaboration [11].

### Limitations

Experiences from the two sites may not be generalizable within China, although they provide common but distinctive discussions around the drivers and barriers to service integration. This study is limited on the discussion of the process of integration, though a concurrent study is being conducted to compare the process and effect of implementing the integrated model with the CDC model in China. This study only focused on the collaboration between the CDC and designated hospital and neglected other important aspects of integration, such as the internal management of the designated hospital, the collaboration between the designated hospital and other general health facilities including primary health care organizations. However, we believe that the relationship between the CDC and designated hospital is the best entry point to understand the issues of integration. This study cannot escape the subjectivity and recall bias inherent qualitative studies. However, efforts have been made to improve the reliability and validity of the study findings.

## Conclusion

The process of integration identifies similarities and differences between sites in more affluent East China and poorer West China. Integration of TB services in the hospitals needs to address the challenges of stakeholder motivations and resource allocation. Effective inter-organizational collaboration could help to improve the efficiency and quality of TB control.

## Competing interests

The authors declare that they have no competing interests.

## Authors' contributions

GZ, XW, JW, QS and JY were involved in conception and design of this project, while GZ, XW, QS and JY were involved with the implementation of the project and analysis and interpretation of the data. GZ and XW have drafted the manuscripts while JW and QS have provided critical comments. All authors read and approved the final manuscript.

## Pre-publication history

The pre-publication history for this paper can be accessed here:

http://www.biomedcentral.com/1472-6963/12/21/prepub
